# A Perspective on Imiquimod Microneedles for Treating Warts

**DOI:** 10.3390/pharmaceutics13050607

**Published:** 2021-04-22

**Authors:** Tsu-Man Chiu, Ping-Chun Hsu, Mohd Yaqub Khan, Cheng-An J. Lin, Chun-Hung Lee, Tsai-Ching Hsu, Min-Hua Chen, Nobutaka Hanagata

**Affiliations:** 1 Department of Dermatology, Changhua Christian Hospital, Changhua County 50094, Taiwan; 68003@cch.org.tw; 2 Department of Biomedical Engineering, Chung Yuan Christian University, Taoyuan City 320314, Taiwan; g10875018@cycu.edu.tw (P.-C.H.); g10802504@cycu.edu.tw (M.Y.K.); chengan_lin@cycu.edu.tw (C.-A.J.L.); bearlee@nhri.edu.tw (C.-H.L.); 3 Institute of Medicine, Chung Shan Medical University, Taichung City 40201, Taiwan; htc@csmu.edu.tw; 4 Institute of Biomedical Engineering and Nanomedicine, National Health Research Institutes, Miaoli County 35053, Taiwan; 5 Clinical Laboratory, Chung Shan Medical University Hospital, Taichung City 40201, Taiwan; 6 Nanotechnology Innovation Station, National Institute for Materials Science, Tsukuba, Ibaraki 3050047, Japan; HANAGATA.Nobutaka@nims.go.jp

**Keywords:** warts, dissolving microneedles, gelatin, imiquimod, immunomodulator

## Abstract

Warts are a common skin problem and are caused by infection with a virus. Warts are currently mainly treated by therapies involving ablating tissue or interrupting cellular division. However, all these existing treatments are either invasive or cause skin pain and tissue destruction. Imiquimod is a synthetic compound that belongs to the imidazoquinolinone family. It has been successfully used as a topical drug to treat external anogenital warts. However, topical imiquimod cream for warts is restricted by low skin permeability, and several side effects such as itching, pain, and erosions occur most frequently following topical treatment. Microneedle technology, a minimally invasive drug delivery system, has the potential to overcome the barrier of the stratum corneum. This technique would also offer a painless treatment choice and provide personalized therapies. In the study, we loaded imiquimod within dissolving microneedles using the molding method. Gelatin was used as a structural material for microneedle formation without adding a crosslinker. To our knowledge, this is the first study of using dissolving microneedles and exploring their utilization with imiquimod for the treatment of warts. First, we added fluorescent dye and trypan blue into the microneedles to evaluate the status of drugs in the microneedles and the degradation property of microneedles made of gelatin, respectively. Here we also prove the strength of the imiquimod microneedles and study their capability to penetrate the skin. The results show no apparent differences in mechanical failure after an additional imiquimod-loaded. Besides, we provide evidence that imiquimod microneedles induce secreted embryonic alkaline phosphatase (SEAP) in the RAW 264.7 macrophages. Gelatin does not affect the imiquimod in microneedles; a similar immune response was affected by the imiquimod alone or imiquimod complexed with gelatin. Our research demonstrates a proof of concept of using imiquimod microneedles for future warts treatment.

## 1. Introduction

Warts are a common disease of the skin caused by infection with a virus. In the survey conducted in school children in Taiwan, 6.9% of children were infected with viral warts, among which 12 to 16 years was the most remarkable age [1]. Current treatments depend upon the ablation of warts (cryotherapy, laser vaporization, electrodesiccation, salicylic acid, silver nitrate, and trichloroacetic acid) or the interruption of the organic process (podofilox, intralesional or systemic interferon, intralesional Bleomycin, and 5-fluorouracil); however, all these treatments are either invasive or cause skin pain and tissue destruction [2,3].

Imiquimod, 1-(2-methyl propyl)-1H-imidazo[4,5-c]quinolin-4-amine (C_14_H_16_N_4_), is a synthetic compound belonging to the imidazoquinolone family of drugs which is suitable for topical treatment and has been successfully used as a topical immune response modifier for the treatment of external anogenital warts [4]. Imiquimod signals the immune system’s innate arm through the Toll-like receptor 7 (TLR7). Once imiquimod is delivered into the epidermal layer of skin, it will lead to antigen-presenting cells’ activation and subsequently lead cells to migrate to local lymph nodes to activate the adaptive immune system [5,6]. However, several side effects have also been observed using imiquimod. Using too much of this medicine or using it for too long can increase the risk of unwanted skin reactions, such as skin itching, pain, erosions, etc. Additionally, patients should avoid sunlight exposure after topically applying the imiquimod [7].

Thus, compared to those invasive and painful methods, microneedle technology, a minimally invasive drug delivery system, can be an alternative to deliver the topical drug through the skin. This technique would offer painless treatment choices and improve patient adherence [8]. Besides, it also provides an opportunity for personalized therapy, especially for the elderly and children [9]. Microneedles are arrays attached to a base-supporting patch, ranging from 25 to 2000 μm in length. They can easily penetrate the stratum corneum’s skin barrier but are short enough to attenuate the invasion and are painless to the patient. Several researchers recently found microneedle vaccine can effectively provide immunization against infectious disease and are as helpful as a syringe used for vaccine injection [8,10].

There are four types of microneedle arrays (solid, coated, dissolving, and hollow) that have been developed to date [11]. Every kind of microneedle has pros and cons. A solid microneedle punctures the skin’s surface and applies it to the skin layer, allowing the drug to diffuse through the holes slowly. The coated microneedle is typically coated with a water-soluble drug. The hollow microneedles are similar to a conventional syringe of short length in shape, allowing liquid medication to be injected directly into the skin layer. However, these microneedles are made of non-biodegradable materials, which could possibly break and be left in the skin to induce a severe inflammatory response [12]. Unlike other types, dissolving microneedles are made of water-soluble materials, allowing cutaneous drug release once the needles come into contact with tissue fluid, and prevent possible disease without leaving a puncture wound [13].

Recently, imiquimod-coated microneedles have been developed [14,15]. They are prepared by a dip-coating method, which is the process of immersing patches in a liquid solution to coat the drug onto the surface of microneedle tips. Although this technique is simple, the procedure requires suitable excipients to stabilize the drug during the coating process and thus reduce its activity. Additionally, these microneedles are non-biodegradable materials, which are at risk in clinical use.

Dissolving microneedles are considered the safest transdermal delivery system mainly because of the selected polymers’ biocompatibility and biodegradation [6]. Numerous polymers have been utilized as structural materials for microneedles vaccine, such as polyvinyl pyrrolidone, polyvinyl alcohol, hyaluronan, and sodium carboxymethyl cellulose, all of which have been investigated [6,16,17].

Gelatin has been approved by the United States Food and Drug Administration as a natural water-soluble polymer and has been reported as an adjuvant for enhancing immunity to vaccines [18]. We think that gelatin as a structural material for microneedles will be better than the above materials.

Here we investigate the imiquimod microneedles prepared using water-dissolvable gelatin. The mechanical and drug delivery properties of imiquimod microneedles and the immune stimulatory effect of imiquimod with gelatin complex were studied in this work. To our knowledge, this is the first study of using dissolving microneedles and exploring their utilization with imiquimod for warts treatment. Many studies add glutaraldehyde as a crosslinker when preparing gelatin microneedles, but it will cause cell toxicity [9]. In contrast, our imiquimod microneedles were prepared without adding a crosslinker. Our research aims to demonstrate a proof of concept of using imiquimod microneedles for future warts treatment.

## 2. Materials and Methods

### 2.1. Preparation of Imiquimod Microneedle Patch

Gelatin solution was prepared by adding gelatin powder (PanReac AppliChem, Barcelona, Spain) to deionized water (with a different weight ratio of gelatin to water for 1:1; 1:1.5; 1:2; and 1:2.5) and heated to 50 °C for 20 min to dissolve the gelatin completely. For preparing imiquimod microneedles, we poured 80 μL of imiquimod (Alfa Aesar, Lancashire, UK) (2 mg/mL; dissolved in dimethyl sulfoxide aqueous solution (DMSO)) into the microneedle mold. The mold is a commercial silicone negative microneedle mold (containing 10 × 10 array with 600 µm in needle height, and 200 × 200 µm in needle base), purchased from Micropoint Technologies Ptr Ltd., Singapore. We then centrifuged the mold for 5 min to compact the imiquimod solution in the mold cavity. After removing the residual imiquimod solution from the mold’s surface, we further poured gelatin solution (80 μL) into the mold. We then centrifuged the mold for 30 min to compact the gelatin polymer in the mold cavity. Finally, we dried the mold at room temperature for one day and obtained the microneedles by removing the silicone mold using adhesive tape.

Gelatin microneedles that did not contain imiquimod were used as a control group. To demonstrate the condition of drugs in microneedles, we used fluorescein isothiocyanate (FITC)-fluorescent dye solution instead of imiquimod solution and followed the same preparation procedure of imiquimod microneedles. All chemicals were of analytical grade and used as received without further purification.

### 2.2. In Vitro Insertion Capability

To understand the penetration and drug release capabilities of microneedles in vitro, we filled the microneedles with trypan blue and then applied them into a porcine cadaver’s skin. To simulate the clinical application situation in the future, we directly pressed the microneedles on the porcine cadaver’s skin with fingers for 1 min, then fixed the microneedles with breathable tape, and analyzed the trypan blue release at different time points (10, 20, 30, 45, and 60 min). After removing the microneedles, we immediately put the porcine skin in liquid nitrogen. We then washed with a phosphate-buffered saline (PBS) solution to remove the trypan blue residual before digital color camera imaging (EC3; Leica, Wetzlar, Germany). The morphology of microneedles and porcine cadaver skin was also assessed using scanning electron microscopy (SEM) (TM3000; Hitachi, Tokyo, Japan). The histological section was prepared by embedding porcine cadaver skin tissue in optimal cutting temperature (OCT) medium and cutting it into 30 μm thick sections using cryotome. Section tissues were attached to slides and analyzed using a confocal laser-scanning microscope (SP5; Leica, Wetzlar, Germany).

### 2.3. Mechanical Strength

In this study, we evaluate the mechanical strength of the needles. The texture analyzer (ME-8236; PASCO, Roseville, CA, USA) was used for measuring the mechanical strength of microneedles. The cylinder stainless steel probe was linked to the top station, and we attach the microneedle patch to the bottom station of the texture analyzer. For each test, the probe was pressed vertically into the microneedle tips and recorded until the needles failed.

### 2.4. In-Vitro Immune Response

We examined the in-vitro effect of imiquimod on immune cell lines. RAW-Blue™ cells (Invivogen, San Diego, CA, USA) are derived from RAW 264.7 macrophages. They strongly express secreted embryonic alkaline phosphatase (SEAP) gene inducible by nuclear factor kappa B (NF-kB) and activator protein 1 (AP-1) transcription factors. Once the Toll-like receptors (TLRs) were stimulated (except TLR5), RAW-Blue™ cells activated NF-kB and/or AP-1, secreting SEAP [7]. To be closer to the actual use of microneedles, we prepared the imiquimod-gelatin complex solution based on the same imiquimod microneedle composition and preparation method described above. The imiquimod-gelatin complex solution was assessed with RAW-Blue™ cells at a final concentration range of 0 to 4 μg/mL in 96-well plates at 37 °C in a 5% CO_2_ incubator. After 21 h of co-culture of the materials with the cells, we removed 50 μL of induced RAW-Blue™ cells supernatant to 150 μL of resuspended QUANTI-Blue™ in a new 96 well plate and baked at 37 °C for another 90 min. The SEAP level was finally determined by measuring the samples’ absorbance using an enzyme-linked immunosorbent assay (ELISA) (Synergy LX; BioTek, Winooski, VT, USA) reader at 630 nm. Imiquimod alone was regarded as the control group.

All of the values above were presented as mean ± standard error of the mean of at least three repeats. Statistical analysis was performed using the analysis student’s paired t-test, comparing two sets of independent groups. Values of * *p* < 0.05 were considered statistically significant.

## 3. Results

### 3.1. Preparing and Characterization of Gelatin Microneedle Patch

**Figure 1:**
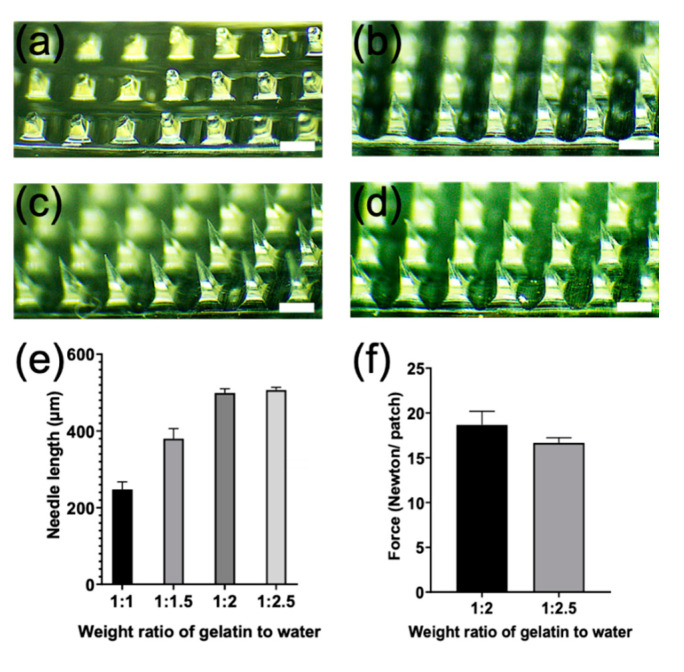
Brightfield microscope images of microneedles arrays. Microneedles were fabricated with a various weight ratio of gelatin to water at (**a**) 1:1; (**b**) 1:1.5; (**c**) 1:2; and (**d**) 1:2.5; (**e**) the needle length of microneedles with various weight ratios of gelatin to water; and (**f**) the mechanical failure of microneedles by comparing the weight ratio of 1:2 with 1:2.5 component of microneedles. Scale bar equals 200 µm.

We made the microneedle patch with gelatin and water without adding a crosslinker, reducing toxicity risk. For making the ideal microneedles, we first assessed the different weight ratios of gelatin to water into a negative mold microneedle array (600 µm in length). The results showed that the weight ratio of 1:2 and 1:2.5 (gelatin to water ration) made it easier to prepare microneedles (around 500 µm in length) (Figure 1a–d), while the other ratios containing lower water caused the gelatin solutions to be too viscous to make microneedles properly (Figure 1a,b). By comparing the weight ratio of 1:2 with 1:2.5 microneedles, we further found that the mechanical strength of the 1:2 component was mildly higher than that of 1:2.5 (Figure 1f). Thus, a gelatin to water ratio of 1:2 component was selected to prepare the microneedles for the following study.

**Figure 2:**
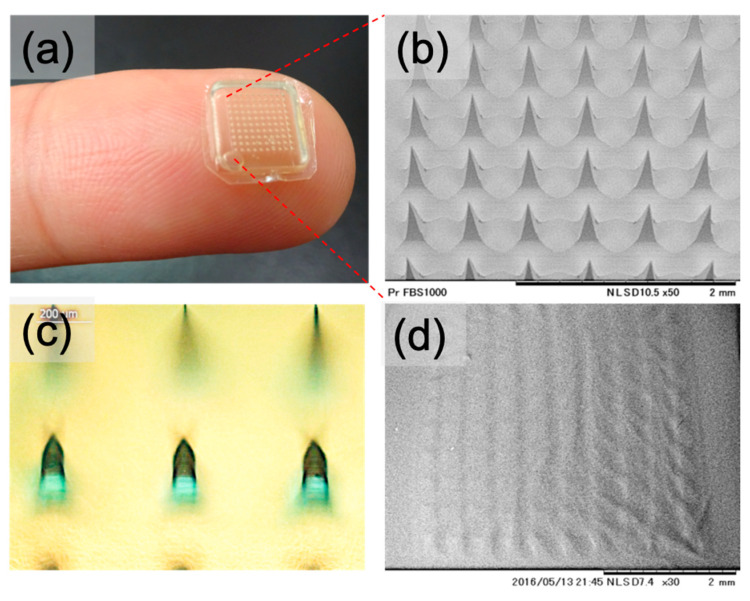
Characteristics of a gelatin microneedle patch. (**a**) The size and shape of the microneedle patch on the fingertip; (**b**) SEM image of microneedle patch with shape 10 × 10 array, around 500 µm needle length, with a total area of 64 mm^2^; (**c**) microneedles with FITC-fluorescent dye loading; and (**d**) the morphology of microneedle tips after placing on the surface of PBS for 3 min.

The gelatin microneedles were uniformly shaped in a 10 × 10 array, around 500 µm needle length, with a total area of 64 mm^2^ (Figure 2a,b). We loaded the FITC-fluorescent dye in the microneedle matrix to understand the drug’s status in microneedles based on the same imiquimod microneedle preparation method. Optical imaging results suggested that the fluorescent dye (dark green color) was mainly deposited at microneedles’ tips (Figure 2c), so we used this method to prepare the imiquimod microneedles. Moreover, the microneedles were dissolved immediately in solution (within 3 min) when put on the PBS surface, indicating this gelatin microneedle patch belongs to dissolving microneedles (Figure 2d).

**Figure 3:**
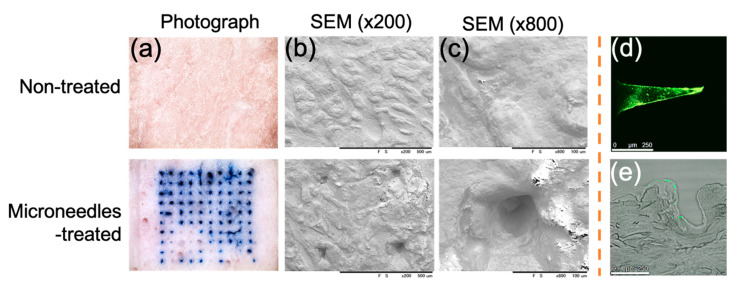
The ability to penetrate the skin in vitro was evaluated by introducing microneedles before and after insertion into the porcine cadaver skin. Skin surface with or without microneedles’ administration was assessed by (**a**) digital color camera; (**b**,**c**) with different scales by using SEM; (**d**) FITC microneedle section, and (**e**) the histological section using a confocal laser-scanning microscope (scale bar equals 250 µm).

The ultimate goal of microneedles is to insert the needles through the skin and achieve effective transdermal drug delivery. Thus, we administered the dye-loaded (trypan blue or FITC-fluorescent) microneedles to the porcine skin to evaluate the ability of the microneedles on the skin tissue. After removing the microneedles, the porcine skin surface was immediately frozen and washed prior to imaging. An image of the skin surface showed a series of blue dots corresponding to the trypan blue-loaded microneedles’ insertion site (Figure 3a). The SEM morphology correctly identified the microneedles’ holes (Figure 3b,c). The histology sections further referred that FITC-fluorescent dye was mainly deposited (dissolved) at the microneedles’ tips (Figure 3d). After the FITC-loaded microneedles were attached to the porcine skin for 1 min, there was a bit of FITC-fluorescent dye residue around the place where the microneedles were inserted (Figure 3e). The depth of microneedle penetrating the tissue was about 500 µm. The penetration depth is quite close to the length of the microneedles. Such penetration depth is sufficient to reach beyond the epidermis layer and into the dermis layers.

**Figure 4:**
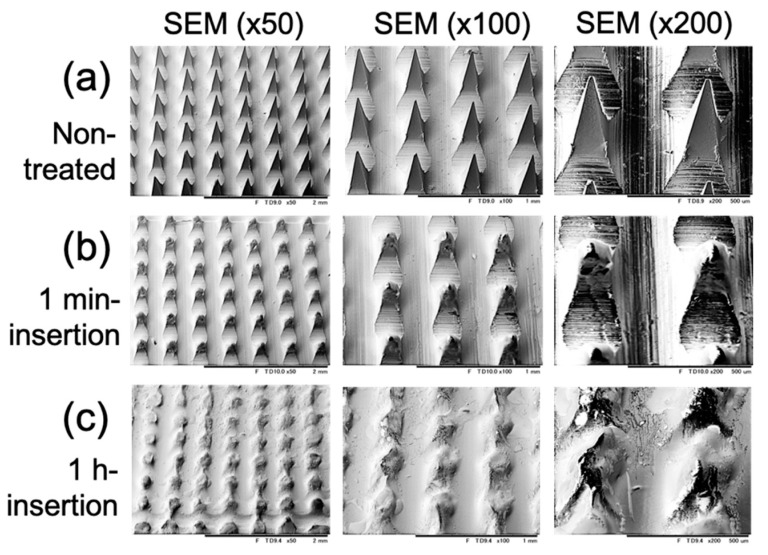
The images of microneedle patch (**a**) before; (**b**) after 1 min; and (**c**) after 1 h of insertion into porcine cadaver skin. The images were performed with different scales by using SEM.

**Figure 5:**
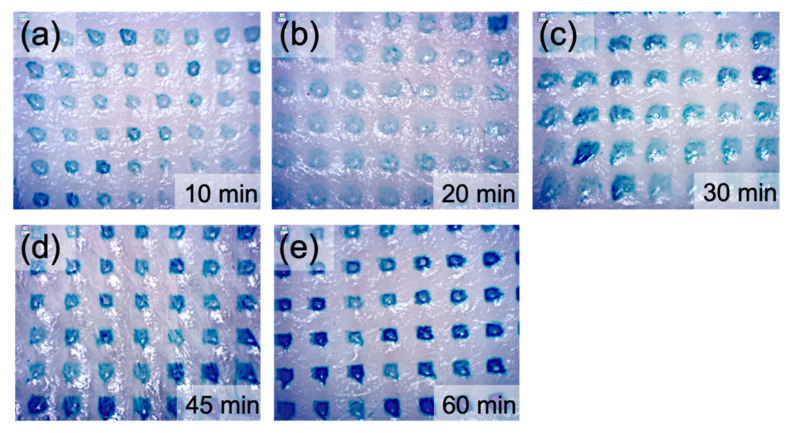
The effect of trypan blue release after the insertion of microneedles into a porcine cadaver skin. The images were performed at different time intervals at (**a**) 10 min; (**b**) 20 min; (**c**) 30 min; (**d**) 45 min; and (**e**) 60 min.

Furthermore, after insertion into the porcine cadaver’s skin, the microneedle surface showed that the small-pointed microneedles were shortened with the prolongation of insertion time (Figure 4). The microneedles do not bend or break on the skin. At the same time, we see the trypan blue gradually released into the insertion hole (Figure 5). Overall, the results confirmed that the gelatin microneedles penetrate the porcine skin and can be administered to facilitate drug release.

### 3.2. Mechanical Strength

Because of the encouraging results from gelatin microneedles, we further studied imiquimod microneedles. Imiquimod is highly insoluble in water. We first dissolved the imiquimod in DMSO and then centrifuged it to compact the imiquimod into the mold cavity. After that, we further poured gelatin solution into the mold to form the imiquimod microneedles. Based on the above experimental procedures, we let the imiquimod homogeneously encapsulate in the microneedles’ tips.

**Figure 6:**
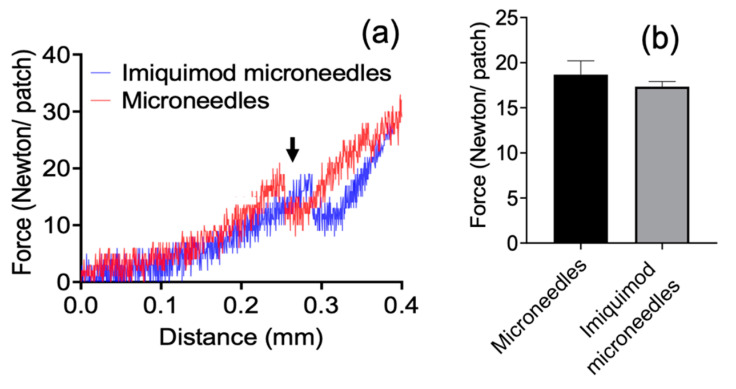
The mechanical failure of gelatin microneedles and with the additional imiquimod-loaded microneedles. The axial failure of imiquimod-loaded microneedles was evaluated by vertically applying forces onto patches. (**a**) the spectrum of microneedles’ mechanical strength; and (**b**) the failure force of microneedles.

The axial failure of imiquimod-loaded microneedles was evaluated by vertically applying forces onto patches. Once the axial load overloaded the microneedles at certain distances from tips (around 300 µm away, as indicated by the arrow), a sharp decrease in mechanical strength was observed due to mechanical failure of needles (Figure 6a). The breaking point appeared at the force of around 17 Newton in each microneedle patch, and this force is equivalent to 0.17 Newton per needle. The results showed no apparent differences in mechanical failure between microneedles and additional imiquimod-loaded microneedles (*p* > 0.05) (Figure 6b). After analysis, we can say that imiquimod did not affect microneedles’ mechanical strength and showed similar resistance to gelatin microneedles.

### 3.3. In Vitro Immune Response

To demonstrate a proof of concept that imiquimod could be used to treat warts, we assessed the effect of imiquimod with gelatin complex on immune response using the RAW-Blue^TM^ cell line. RAW-Blue cells, derived from RAW264.7 macrophages, have been widely reported and can be used to study signal transduction pathways by imiquimod activations [7]. We co-cultured the imiquimod-gelatin complex solution with the cells. Once TLRs (except TLR5) are stimulated, RAW-Blue cells will activate NF-κB and/or AP-1, leading to the SEAP gene induction, which could be detectable under the QUANTI-Blue^TM^ kit.

**Figure 7:**
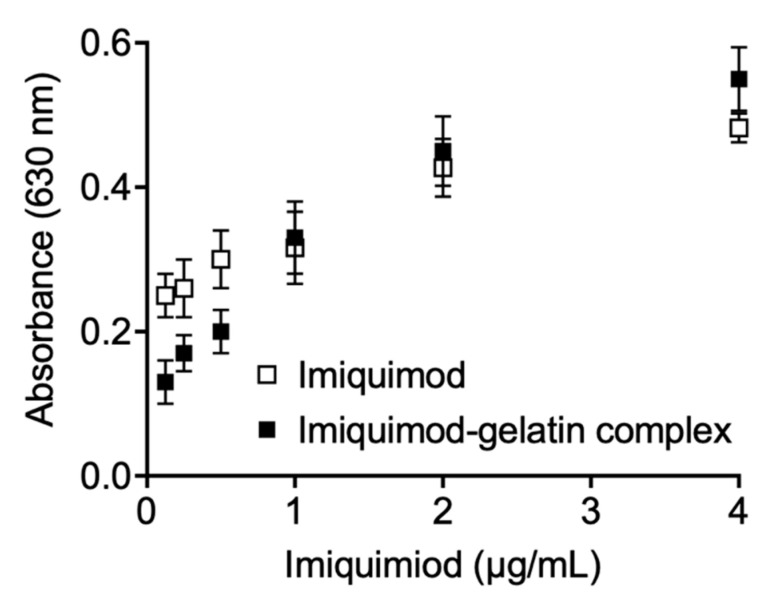
The effect of imiquimod and imiquimod-gelatin complex solution on immune response using RAW-BlueTM cell line. The induction of SEAP level was determined by measuring the samples’ absorbance at 630 nm.

The imiquimod-gelatin complex was prepared according to the microneedles’ procedure and assessed at various concentrations on the RAW-Blue cell line. The results showed that gelatin alone did not affect SEAP induction (data not shown). When the imiquimod-gelatin complex solution was treated on cells, SEAP-fluorescence increased with increasing the dose compared with gelatin alone (Figure 7). The imiquimod and imiquimod-gelatin complex’s fluorescence values did not show a significant difference (*p* > 0.05). Statistical analysis was performed using the analysis student’s paired t-test, comparing two sets of independent groups of curves. The results suggest that whether imiquimod is delivered alone or in the form of microneedles made of gelatin, a similar immune response will be affected by the imiquimod on the cell line. However, when we further confirmed at low imiquimod concentration (below 1 µg/mL), we found that imiquimod delivered alone is significantly higher than imiquimod–gelatin complex. On the contrary, when at a higher concentration (4 µg/mL), the imiquimod–gelatin complex is significantly higher than the imiquimod delivered alone. We speculate that this phenomenon may be related to the concentration of gelatin polymer. It has been reported to contribute to enhancing cell uptake and immune stimulation [19].

## 4. Discussion

We used gelatin polymer as a structural material to make dissolving microneedle structures achieve clinical applications. According to 501 (a)(2)(B) of the Federal Food, Drug, and Cosmetic Act (FD&C Act), soluble microneedles derived from a soluble polymer complex are classified as a “drug product” [20]. Therefore, these soluble substances’ safety, biochemistry, and decomposition products should be considered. Gelatin holds promise in this regard, as it has been approved by the United States Food and Drug Administration as a natural water-soluble polymer and has been used as drug capsules for several years. Furthermore, it has been reported that gelatin is safe to use as a stabilizer in a vaccine mixture and contributes to enhancing cell uptake and immune stimulation [19]. Because gelatin has the characteristics of biocompatibility, biodegradation, and low immunity nature, we choose it as a structural material for microneedle formation.

The thickness of the outermost of the skin, the epidermis layer, is around 200 µm and almost 25% of the immune cells (such as dendritic cells, Langerhans cells, and other antigen-presenting cells) grow in the entire skin area [21]. The length of gelatin microneedles (approximately 500 µm) is advantageous for penetration through the stratum corneum layer. It is still short enough to minimize patient invasiveness and pain associated with syringe injection (Figure 1c,e). Thus, these gelatin microneedles are adequate for delivering the imiquimod into the sub-epidermal layer and targeting antigen-presenting cells. These cells can migrate to the lymph nodes and elicit an immune response.

For the treatment of warts, imiquimod can be encapsulated in the microneedles or coated on their surface. Bleomycin, a glycopeptide antibiotic, was reported to be coated on microneedles’ surface to treat warts [14]. However, coated microneedles require suitable excipients to stabilize the drug during the coating process and reduce the drug’s activity. The procedure also has the drawback of drug wastage and loss, the variable coating thickness of active ingredients onto microneedles, and inaccuracy in drug dosage [22]. In contrast, imiquimod is dissolved in the DMSO solution and then encapsulated in dissolving microneedles using gelatin polymer in our study. DMSO can help dissolve the imiquimod in the gelatin and be regarded as a pharmaceutical penetration enhancer facilitating transdermal drug delivery [23]. Here we made the microneedle patch with gelatin polymer without adding a crosslinker. Many studies add glutaraldehyde as a crosslinker when preparing gelatin microneedles to enhance gelatin’s mechanical strength, but it will cause cell toxicity [9,24]. Compared with non-degradable microneedles, although gelatin microneedles have weaker mechanical strength, they can be designed to degrade in tissue to control drug release. These dissolving microneedles would be more adapted for children’s treatment, where microneedles can be inserted, removed, and discarded without making the patient wait. There are also no bio-hazardous sharp wastes after use [6].

For the mechanical strength, each needle of imiquimod-loaded microneedles can resist a force of around 0.17 Newton (Figure 6). Compared with other studies, Davis et al. [25] suggested that microneedles generally required 0.08 to 3.04 Newton to insert them into the skin. However, the force is varied depending on the sharpness, height, aspect ratio, and geometry of the tips [26]. E.Z. Loizidou et al. [26] used microneedles made of sugar and noted that the microneedles were likely to buckle when the applied axial force exceeded 0.04 Newton, even though those sugar microneedles still can be used to puncture the skin under this force. As such, the study of the in vitro insertion becomes more critical than mechanical strength in determining whether the microneedles will effectively penetrate when applied to the skin. In the in vitro study, gelatin microneedles provide high enough mechanical stress for transdermal administration, but they can also degrade rapidly in the skin after one hour of administration (Figure 3 and Figure 4). Furthermore, after insertion into the porcine cadaver’s skin, the microneedles showed that the trypan blue gradually released into the insertion hole, indicating gelatin structure material will be adequate for controlling drug delivery (Figure 5).

Furthermore, we show a proof of concept from in vitro studies that imiquimod-loaded microneedles dissolved in the skin can activate macrophages expressing SEAP (Figure 6). Once the Toll-like receptor was stimulated, RAW-Blue™ cells activated NF-kB and/or AP-1, secreting SEAP [7]. These results suggest that imiquimod microneedles can activate macrophages via TLR7 to secrete pro-inflammatory cytokines (primarily interferon-α (IFN-α), interleukin-6 (IL-6), and tumor necrosis factor-α (TNF-α)), and finally effectively enhance adaptive immune response [27]. However, the most significant obstacle is that the skin’s barrier function, exerted by the stratum corneum, impairs this immunomodulator’s penetration and absorption.

The 5% *w*/*w* imiquimod cream (Aldara^TM^) is licensed for the topical treatment of warts. However, topical imiquimod cream for warts is restricted by low skin permeability. According to a report by I Telò et al. [28], as the imiquimod solution (dissolved in DMSO) is applied to the skin, the maximum accumulation amount in the skin is around 1 µg/cm^2^, even though it has also been found that only 19% of the applied imiquimod cream can remain on the skin after topical treatment [29,30]. Therefore, although our prepared microneedle patch can only contain about 0.3 µg (equal to 0.46 µg/cm^2^) of imiquimod at most, they will be more clinically beneficial than the current imiquimod cream. Based on microneedle technology, imiquimod can be delivered in the sub-epidermal layer, thus activating antigen-presenting cells and activating T cells. Then, B cells are activated by T cells, resulting in a robust adaptive immune response against warts. Moreover, microneedles can also reduce pain and increase patient adherence compared to other invasive methods like cryotherapy, electrocautery, surgical excision, and laser. Based on the above study, we find that imiquimod microneedles can be one of the best options to treat warts, especially in non-genital areas.

## 5. Conclusions

The study has demonstrated a proof of concept as a microneedle-based imiquimod delivery system to treat warts in the future. According to our preliminary finding, microneedles can be formed from gelatin without adding a crosslinker. The needles were obtained in microscale (10 × 10 array, approximately 500 µm in height, with a total area of 64 mm^2^). The mechanical strength of microneedles is high enough to penetrate through the epidermis. The immune study, demonstrated by RAW-Blue™ cells, revealed that the imiquimod and imiquimod-gelatin complex did not show a significant difference. The results suggest that whether imiquimod is delivered alone or in the form of microneedles made of gelatin, a similar activation on the immune cells will be achieved. Thus, imiquimod microneedles may be one of the best options to treat warts in non-genital areas. For further use in clinical practice, we need to do more study on papillomavirus infections in animal models.

## Data Availability

Not applicable.
